# Comparing the short-term outcomes of intracorporeal esophagojejunostomy with extracorporeal esophagojejunostomy after laparoscopic total gastrectomy for gastric cancer

**DOI:** 10.1186/s12893-016-0130-9

**Published:** 2016-03-21

**Authors:** Ke Chen, Yang He, Jia-Qin Cai, Yu Pan, Di Wu, Ding-Wei Chen, Jia-Fei Yan, Hendi Maher, Yi-Ping Mou

**Affiliations:** Department of General Surgery, Sir Run Run Shaw Hospital, School of Medicine, Zhejiang University, 3 East Qingchun Road, Hangzhou, 310016 Zhejiang Province China

**Keywords:** Laparoscopic gastrectomy, Intracorporeal anastomosis, Hand-sewn, Stomach neoplasms

## Abstract

**Background:**

Totally laparoscopic distal gastrectomy (TLDG) using intracorporeal anastomosis has gradually developed due to advancements in laparoscopic surgical instruments. However, totally laparoscopic total gastrectomy (TLTG) with intracorporeal esophagojejunostomy (IE) is still uncommon because of technical difficulties. Herein, we evaluated various types of IE after TLTG in terms of the technical aspects. We compared the short-term operative outcomes between TLTG with IE and laparoscopy-assisted total gastrectomy (LATG) with extracorporeal esophagojejunostomy (EE).

**Methods:**

Between March 2006 and December 2014, a total of 213 patients with gastric cancer underwent TLTG and LATG. Overall, 92 patients underwent TLTG with IE, and 121 patients underwent LATG with EE. Generally, there are two methods of IE: mechanical staplers (circular or linear staplers) and hand-sewn sutures. Surgical efficiencies and outcomes were compared between two groups. We also described various types of IE using a subgroup analysis.

**Results:**

The mean operation times were similar in the two groups, as was the number of retrieved lymph nodes. However, the mean estimated blood loss of TLTG was statistically lower than LATG. There were no significant differences in time to first flatus, the time to restart oral intake, the length of the hospital stay after operation, and postoperative complications. Four types of IE have been applied after TLTG, including 42 cases of hand-sewn IE. The overall mean operation time and the mean anastomotic time in TLTG were 279.5 ± 38.4 min and 52.6 ± 18.9 min respectively. There was no case of conversion to open procedure. Postoperative complication occurred in 16 patients (17.4 %) and no postoperative mortality occurred.

**Conclusions:**

IE is a feasible procedure and can be safely performed for TLTG with the proper laparoscopic expertise. It is technically feasible to perform hand-sewn IE after TLTG, which can reduce the cost of the laparoscopic procedure.

## Background

Gastric cancer is the second leading cause of cancer related deaths and the fourth most common form of cancer worldwide. Nearly 70 % of all new cases and deaths occur in developing countries, and about 40 % of those occur in Eastern Asia [1, 2]. Since the introduction of laparoscopy-assisted distal gastrectomy (LADG) for gastric cancer in 1994 [3], LADG has become widely used for tumors located in the lower stomach with satisfactory surgical outcomes. However, the inclusion of the auxiliary incision in LADG makes it divergent from the minimally invasive treatment concept pursued in minimally invasive surgery. During the past decade, some studies have demonstrated the safety and feasibility of totally laparoscopic gastrectomy with intracorporeal reconstruction [4–6]. We have also reported that totally laparoscopic distal gastrectomy (TLDG) with intracorporeal reconstruction is better than laparoscopy-assisted distal gastrectomy (LADG) with extracorporeal reconstruction as it has improved outcomes such as better cosmesis, earlier bowel movements, less pain, and shorter hospital stays [7, 8]. These advantages are attributed to the less invasiveness of totally laparoscopic surgery. However, regarding to the laparoscopic total gastrectomy (LTG), many surgeons have preferred the “laparoscopy-assisted” type because of the high technical demand of intracorporeal esophagojejunostomy (IE). In addition to the relatively low incidence of upper gastric carcinoma in East Asia [2], TLTG for upper and middle gastric cancer has not been generalized. As the development of laparoscopic instruments, various types of IE using linear or circular staplers have recently been reported [4]. A growing number of surgeons are beginning to pay attention to TLTG and accept it. Nevertheless, to the best of our knowledge, there have been no studies that clarify the best approach. On the basis of our laparoscopic experience gained from laparoscopy gastric and pancreatic surgery, and other laparoscopic operations [7–14], we were encouraged to develop TLTG with various types of IE for the treatment of upper and middle gastric cancer. In this article, we present our experiences and short-term clinical outcomes of TLTG with various types of IE using laparoscopic staplers or hand-sewn purse-string suture technique. We also compare those outcomes of patients with laparoscopy-assisted total gastrectomy (LATG) from our center to further clarify the safety and feasibility of IE.

## Methods

### Patients

The patients in this research come from the gastric cancer database from March 2006 to December 2014 in the Department of General Surgery. A total of 213 patients underwent LTG with Roux-en-Y reconstruction for gastric cancer. During this period, patients were divided into two groups according to reconstructive methods, such as intra-corporeal or extra-corporeal reconstruction. All of these patients were diagnosed with gastric adenocarcinoma in the upper and middle stomach before surgery and they underwent LTG with modified D2 lymph node dissection. American Joint Committee on Cancer (seventh edition) and TNM classification serve as the criterion for clinical and pathologic staging. This research has been approved by the Zhejiang University’s Ethics Committee. Informed consent was obtained from each patient preoperatively after they were given a detailed explanation of the two procedures-TLTG and LATG. All of the patients agreed to participate in the study.

### Surgical procedure

Our previous essays issued before have elaborated on the lymphadenectomy in detail. With the patient in the supine position, mobilization of the stomach and en bloc systematic lymph node dissection were performed via five trocars under a pneumoperitoneum. Based on the Gastric Cancer Treatment Guidelines 2010 by the Japanese Gastric Cancer Association, which contained not only number D_1_ dissection but also number 7, 8, 9, 10, 11p, 11d, and 12a dissection, lymphadenectomy was conducted.

### Methods of IE

**Fig. 1:**
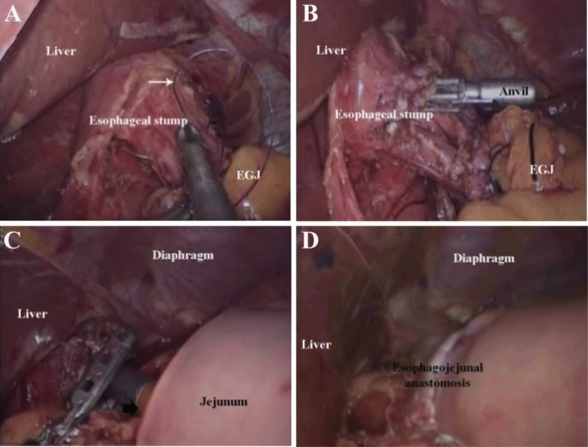
Conventional circular stapler-anvil method. **a** The purse-string suture (white arrow) was placed in the esophagus. **b** The anvil was introduced into the esophageal stump through the hole. **c** The circular stapler was introduced into the jejunum through the jejunal stump and attached with the anvil. **d** The circular stapler was fired and the esophagojejunostomy was completed

1. Conventional circular stapler-anvil approach (Type A group) :The stomach was pulled up and a purse-string suture was located at 1 cm above the incision line, which was decided in advance (Figs. 1a and 2a). The harmonic scalpel made a hole at the esophagogastric junction. The anvil was placed in the esophageal stump through the hole. It was then sewn up with the purse-string suture (Figs. 1b and 2b). Then, esophagogastric junction was separated and the stomach was removed. The circular stapler was placed in the jejunum via the jejunal stump and adhered to the anvil (Figs. 1c and 2c). The esophagojejunostomy was finished after the circular stapler was heated (Fig. 1d). Finally, the jejunal stump was closed with endoscopic linear staplers (Fig. 2d).

**Fig. 2:**
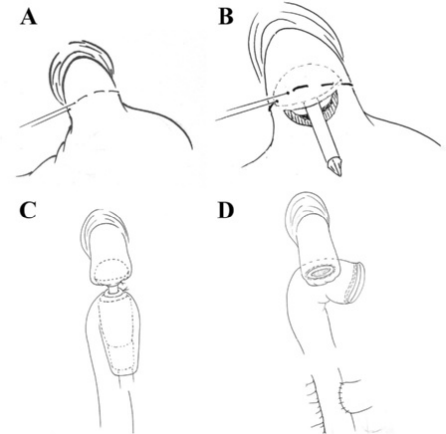
Conventional circular stapler-anvil method (schematic diagram). **a** The purse-string suture (white arrow) was placed in the esophagus. **b** The anvil was introduced into the esophageal stump through the hole. **c** The circular stapler was introduced into the jejunum through the jejunal stump and attached with the anvil.** d** The circular stapler was fired and the esophagojejunostomy was completed
2. Linear stapler side-to-side approach (Type B group): On the end of the jejunum, a small opening was made 10 cm away from the stump and the stump was subsequently extended to the esophagus, where there was a small opening on one side. A side-to-side antiperistaltic esophagojejunostomy was carried out subsequently with linear staplers (Figs. 3a and 4a, b). Finally, the entry hole and esophagus were closed with staplers (Figs. 3b and 4c, d).

**Fig. 3:**
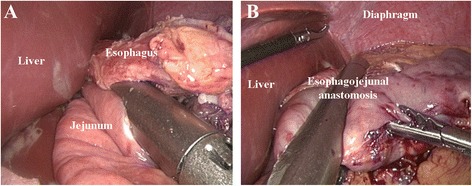
Linear stapler side-to-side method. **a** Each jaw of the linear stapler was inserted into the holes on the esophageal stump and the jejunum and then the linear stapler was fired. **b** The entry hole and esophagus were closed using the stapler

**Fig. 4:**
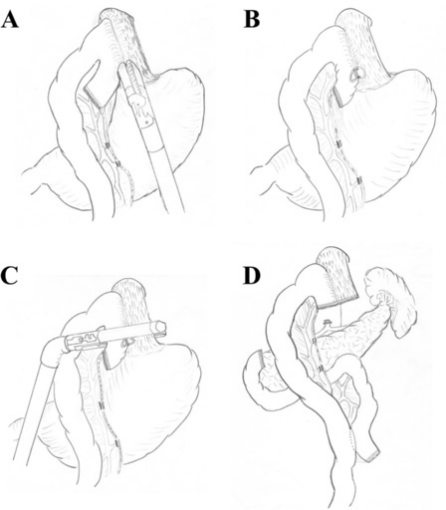
Linear stapler side-to-side method (schematic diagram). **a** and **b** Each jaw of the linear stapler was inserted into the holes on the esophageal stump and the jejunum and then the linear stapler was fired. **c** and **d** The entry hole and esophagus were closed using the stapler
3. Linear stapler delta-shaped approach (Type C group): An endoscopic linear stapler divided the esophagogastric junction and made several small holes on the margin of the esophageal stump and the jejunum. The rear walls of the esophageal stump and the jejunum approached each other and were then connected by the endoscopic linear stapler (Figs. 5a and 6a). Subsequently, the staple line was examined for any potential faults and hemostasis was confirmed. The ordinary opening was pulled up with stay sutures (Figs. 5b and 6b), and was closed with two applications of the linear stapler (Figs. 5c and 6c), leading to the reconstruction of the intracorporeal alimentary tract (Figs. 5d and 6d).

**Fig. 5:**
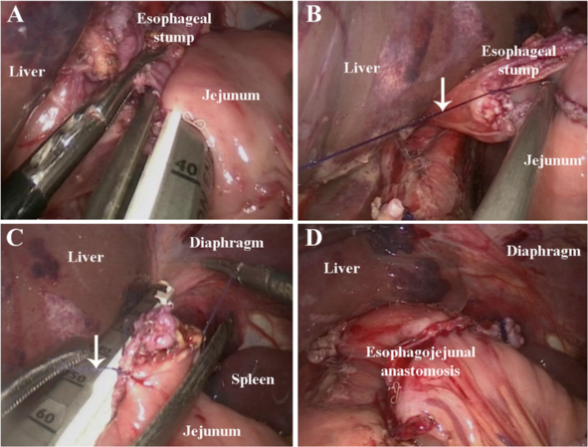
Linear stapler delta-shaped method. **a** Small holes were created along the edge of the esophageal stump and the jejunum which were approximated and joined with the endoscopic linear stapler. **b** Stay sutures (white arrow) were placed to lift the common opening. **c** The common opening was then closed with two applications of the linear stapler. **d** Reconstruction of the intracorporeal alimentary tract was completed

**Fig. 6:**
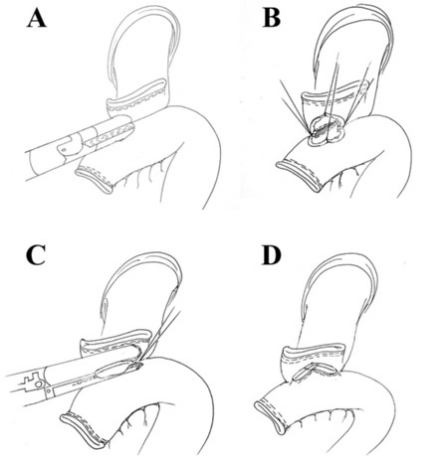
Linear stapler delta-shaped method (schematic diagram). **a** Small holes were created along the edge of the esophageal stump and the jejunum which were approximated and joined with the endoscopic linear stapler. **b **Stay sutures (white arrow) were placed to lift the common opening. **c** The common opening was then closed with two applications of the linear stapler. **d** Reconstruction of the intracorporeal alimentary tract was completed
4. Hand-sewn end-to-side approach (Type D group): The jejunal loop was introduced to approach the esophageal stump. The jejunum was attached to the esophageal stump with several serosal muscularis interrupted sutures, which are located at the rear part of the esophageal stump (Figs. 7a and 8a). During this process, one hole was made on the anti-mesenteric side of the jejunum and the other hole was made on the esophageal stump (Fig. 8b). Several full-thickness continuous sutures were used to sew up the posterior wall (Figs. 7b and 8c). Then, a full-thickness continuous suture closed the anterior wall (Figs. 7c and 8d). Interrupted sutures reinforced the seromuscular layer in order to reduce pressure. (Fig. 7d).

**Fig. 7:**
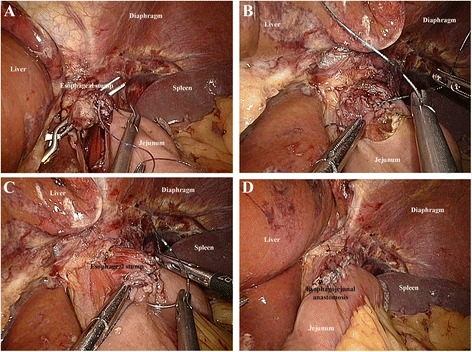
Hand-sewn end-to-side method. **a**: The jejunum was anchored to the esophageal stump by several serosal muscularis interrupted sutures placed in the posterior layer of the esophageal stump. **b**: Several full-thickness interrupted sutures closed the posterior wall. **c**: A full-thickness continuous suture carried out the closure of the anterior wall. **d**: The seromuscular layer was strengthened with interrupted sutures to reduce tension

**Fig. 8:**
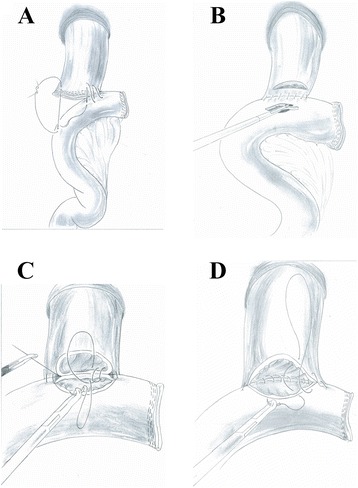
Hand-sewn end-to-side method (schematic diagram). **a** The jejunum was anchored to the esophageal stump by several serosal muscularis interrupted sutures placed in the posterior layer of the esophageal stump. **b** Make an incision in the esophagus and jejunum stump respectively. **c** Several full-thickness interrupted sutures closed the posterior wall. **d** A full-thickness continuous suture carried out the closure of the anterior wall

### Postoperative management

All of the patients stayed in the general ward after surgery. The nasogastric tube was removed at the end of the case in the operating room. Before patients could tolerate a liquid diet, they relied on total parenteral nutrition (TPN). When patients were able to tolerate a liquid diet, they were gradually given a semiliquid diet. In order to be discharged from the hospital patients had to adapt to a semiliquid diet, have a normal blood work panel and temperature, and could not suffer from obvious discomfort. Follow-ups were conducted every 3 months for 2 years, every 6 months for the following 3 years. Most patient’s regular follow-ups included a physical examination, laboratory tests (including CA19-9, CA72-4, and CEA levels), chest radiography, ultrasonography or CT, and endoscopy. All patients were checked on for the rest of their lives or until June 30, 2015, when the follow-up ends.

### Statistical analysis

All statistical analyses were performed using the Statistical Package for the Social Sciences (SPSS®) version 18.0 (SPSS, Inc. Chicago, IL, United States). The differences in the measurement data were compared using the Student’s t test, and comparisons between groups were tested using the χ 2 test or the Fisher exact probability test. *P* < 0.05 was considered statistically significant.

## Results

### Comparison of the clinicopathologic characteristics

There was no conversion to an open procedure, and all procedures were completed under the given conditions. Demographics and clinicopathological characteristics are listed in Table 1. Of the 213 patients, 92 underwent TLTG, and 121 patients underwent LATG. There were no significant differences in age, gender, body mass index, the American Society of Anesthesiologists (ASA) physical status classification, presence of comorbid disease, or tumor stage.

**Table 1:** Comparison of the clinicopathological characteristics

		LATG (*n* = 121)	TLTG (*n* = 92)	*P* value
Age (years)		59.8 ± 11.3	58.7 ± 10.7	0.47
Gender	Male	88	62	0.40
	Female	33	30	
BMI index (kg/m^2^)		22.8 ± 3.1	23.0 ± 3.2	0.65
Comorbidity	Absence	80	65	0.48
	Presence	41	27	
ASA classification	I	63	54	0.49
	II	53	33	
	III	5	5	
Tumor size (cm)		3.4 ± 1.7	3.7 ± 1.9	0.23
Histology	Differentiated	70	59	0.35
	Undifferentiated	51	33	
TNM stage	IA/IB	51/23	25/21	0.25
	IIA/IIB	15/7	12/11	
	IIIA/IIIB/IIIC	7/8/10	12/5/6	

### Comparison of surgical and postoperative outcomes

The outcomes of the operative procedures and postoperative recovery are listed in Table 2. The mean operation time was similar (225.2 ± 41.5 min vs. 220.3 ± 43.5 min, *P* = 0.40) in both groups, but the estimated blood loss of TLTG was statistically lower than LATG (153.1 ± 57.3 mL vs. 132.3 ± 60.4 mL, *P* = 0.01). The proximal margin distance and number of retrieved lymph nodes were not significantly different between the two groups. The time to first flatus was similar between the groups (3.3 ± 1.1 d vs. 3.5 ± 1.1 d, *P* = 0.19), as was the time to restart oral intake after surgery (4.6 ± 1.2 d vs. 4.7 ± 1.3, *P* = 0.56). There was also no difference in the length of the hospital stay after surgery (9.7 ± 2.36 d vs. 9.5 ± 3.3, *P* = 0.60).

**Table 2:** Comparison of surgical outcomes and postoperative recovery

	LATG (*n* = 121)	TLTG (*n* = 92)	*P* value
Operation time (min)	225.2 ± 41.5	220.3 ± 43.5	0.40
Blood loss (mL)	153.1 ± 57.3	132.3 ± 60.4	0.01
Number of retrieved lymph nodes	28.7 ± 7.5	29.9 ± 7.6	0.25
Proximal resection margin (cm)	4.6 ± 1.4	4.9 ± 1.5	0.13
Time to first flatus (days)	3.3 ± 1.1	3.5 ± 1.1	0.19
Time to starting oral intake (days)	4.6 ± 1.2	4.7 ± 1.3	0.56
Postoperative hospital stay (days)	9.7 ± 2.3	9.5 ± 3.3	0.60

The postoperative complications are listed in Table 3. There was no in-hospital mortality and 30-d mortality. Complications developed in 23 (19.0 %) of patients in the LATG group and 16 (17.4 %) of patients in the TLTG group. There was no significant difference between the two groups regarding the postoperative morbidity. One patient in the LATG group underwent reoperation due to anastomotic leakage. Three patients in the TLTG group underwent reoperation, one for anastomotic leakage, one for anastomotic stricture, and one for intracorporeal hemorrhage.

**Table 3:** Comparison of postoperative complications

Variable	LATG (*n* = 121)	TLTG (*n* = 92)	*P* value
Total complication	23	16	0.76
Anastomotic leakage	1	1	1.00
Anastomotic stricture	2	3	0.65
Intracorporeal hemorrhage	1	2	0.58
Abdominal abscess	4	1	0.39
Pulmonary infection	3	1	0.64
Stasis	3	2	1.00
Pancreatic leakage	2	1	1.00
Ileus	3	1	0.64
Lymphorrhea	1	1	1.00
Wound infection	3	1	0.64
Pulmonary embolism	0	1	0.43
Reoperation	1	3	0.32
Mortality	0	0	

### Subgroup analysis for patients who underwent TLTG

The types of anastomotic methods were type A in 18 patients, type B in 22 patients, type C in 10 patients and type D in 42 patients. The mean anastomotic time was 57.5 ± 18.5, 40.0 ± 11.2, 39.0 ± 3.9 and 60.7 ± 17.5 min for these four groups respectively. Intraoperative blood loss, number of retrieved lymph nodes, and postoperative recovery were similar among the four groups. Five patients in type A group, five in type B group, two in type C group, and four in type D group had postoperative morbidity. The operative findings and subsequent postoperative clinical course data are shown in Tables 4 and 5.

**Table 4:** Surgical outcomes of 92 patients who underwent TLTG

	Type A (*n* = 18)	Type B (*n* = 22)	Type C (*n* = 10)	Type D (*n* =42)	Total (*n* = 92)
Operation time (min)	305.6 ± 45.9 (250–380)	266.8 ± 38.7 (230–360)	278.0 ± 16.2 (250–300)	285.4 ± 36.1 (240–420)	279.5 ± 38.4 (230–420)
Anastomotic time (min)	57.5 ± 18.5 (35–90)	40.0 ± 11.2 (25–60)	39.0 ± 3.9 (35–45)	60.7 ± 17.5 (45–105)	52.6 ± 18.9 (25–105)
Blood loss (mL)	80.6 ± 29.4 (50–160)	86.4 ± 39.7 (50–200)	87.0 ± 24.5 (50–120)	82.6 ± 33.7 (50–180)	83.1 ± 33.2 (50–200)
Retrieved lymph nodes	30.9 ± 5.8 (25–45)	34.6 ± 4.1 (25–42)	34.8 ± 6.1 (28–47)	36.1 ± 13.7 (24–69)	35.6 ± 8.9 (24–69)
First flatus (day)	4.2 ± 0.8 (3–5)	3.6 ± 1.3 (2–7)	3.4 ± 0.8 (2–5)	3.5 ± 0.7 (2–5)	3.7 ± 0.9 (2–7)
Liquid diet (days)	5.2 ± 0.8 (4–6)	4.9 ± 1.1 (3–7)	4.6 ± 0.7 (4–6)	4.5 ± 0.9 (3–7)	4.8 ± 0.9 (3–7)
Soft diet (days)	6.7 ± 1.3 (5–11)	6.3 ± 1.1 (5–8)	6.6 ± 0.8 (5–8)	6.5 ± 2.0 (5–15)	6.6 ± 1.5 (5–15)
Postoperative hospital stay (days)	10.9 ± 2.9 (9–20)	10.2 ± 2.4 (8–17)	10.1 ± 2.9 (8–18)	9.2 ± 1.5 (7–17)	10.0 ± 2.3 (7–20)

Data are means ± standard deviations (range)

**Table 5:** Postoperative complication of 92 patients who underwent TLTG

	Type A (*n* = 18)	Type B (*n* = 22)	Type C (*n* = 10)	Type D (*n* =42)	Total (*n* = 92)
Postoperative complication	5	5	2	4	16
anastomotic leakage	1				
anastomosis stricture	1	2			
intracorporeal hemorrhage		1	1	1	
stasis	1		1		
lymphorrhea	1				
pulmonary embolism	1				
abdominal abscess		1			
pulmonary infection		1			
ileus				1	
pancreatic leakage					1
wound infection					1

## Discussion

Laparoscopic-assisted total gastrectomy (LATG) is the most common type of laparoscopic total gastrectomy (LTG), in which lymph nodes are removed with the aid of a laparoscope. Then in order to promote the resection of the specimen and the reconstruction of the digestive tract, an epigastrium assistant incision is created. Totally laparoscopic total gastrectomy (TLTG) is another type of LTG, without extra incisions or touching of the tumor. It reduces the traumatic stress of surgical patients, as it only involves trocar wounds. In the beginning we wished to overcome the drawbacks of cumbersome reconstruction by adopting extracorporeal anastomosis. In 2007, we thought we could improve the surgery procedure, so we started to perform intracorporeal anastomosis followed by LTG. The security and feasibility of both TLTG and LATG in the treatment of gastric cancer located in upper and middle stomach are verified by the research.

LATG is the most commonly used version of LTG. Compared to traditional open gastrectomy, most studies have reported that LATG can achieve better cosmesis, shorter hospital stays and faster postoperative recovery [15–18]. Because the reconstruction step of TLTG is tricky, operating safety is a continuing worry for surgeons. In our study, the operation time of TLTG was slightly shorter than that of LATG and the intraoperative blood loss of the TLTG group was less than that in the LATG group. Those differences might have been due to the time required for mini-laparotomy, which is time-consuming. Also, anastomosis through small skin incisions created by hand manipulation may increase blood loss. However, considering that LATG was performed during the early period of the surgeon’s experience and TLTG was performed during the late period, this time difference appears to be acceptable. Our results also revealed that the overall complications were similar between the two groups. Thus, we believe laparoscopic surgeons with ample experience could be able to achieve a safe and effective digestive tract reconstruction using the TLTG method with a complication rate comparable to that observed with LATG.

According to our experience, TLTG is different from LATG in many ways. First of all, intracorporeal reconstruction adopting endoscopic staplers or hand-sewing techniques can achieve a tension-free anastomosis and reduce unnecessary damage to the surrounding tissue. Secondly, TLTG can be described as a “no touch tumor” operation. It avoids the direct contact and extrusion of tumors. The advantages of this method are that it decreases or avoids stimulation of the lesion and conforms to the principles of tumor-free technique and non-touch radical excision of gastric cancer. Thirdly, TLTG requires a small incision instead of the minilaparotomy. In the case of LATG, there is always an auxiliary 6–7 cm incision under the xiphoid. For obese patients, the incision may be as long as 8–10 cm. By contrast, in the case of TLTG, with the soft hypogastrium wall, the physician can simply broaden the incision for the 10 mm trocar under the umbilicus to a 3–4 cm semicircle incision near the navel so that the sample can be extracted in an appropriate way. On the one hand, the smaller incisions would be less traumatic and less invasive, on the other hand, it avoids the difficulty in reconstruction of anastomosis due to limited operation field especially for obese patients [19].

In our study, we have adopted two methods of IE, including mechanical staples and the hand-sewn suture technique. However, the mechanical anastomosis presented many technical problems including exposure difficulties, impossible reinforced suturing variation in the diameter of the esophagus, and a weak point in double stapling [20, 21]. Due to the technical difficulties of laparoscopic anastomosis and concern regarding anastomotic complications using the stapling method, we were encouraged to use the intracorporeal hand-sewn end-to-side esophagojejunostomy. In our study, 42 patients underwent the intracorporeal hand-sewn esophagojejunostomy. For this subgroup the mean operation time is 285.4 min with a mean blood loss of 82.6 ml. The time to the first flatus and oral intake were 3.5 days and 4.8 days, and the mean postoperative hospital stay was 10.0 days. A recent literature review provided the surgical results of TLTG [22]. The mean surgical time and mean blood loss were 254.2 min and 114.0 ml. The time to the first flatus and time to restart oral intake were 3.3 days and 5.0 days, and the mean postoperative hospital stay was 12.0 days. From the comparison and our acceptable results of mortality and complication rate, the fact that the intracorporeal hand-sewn esophagojejunostomy after TLTG is safe and feasible has been verified. However, when using hand-sewn method, the surgeon has to be skilled at laparoscopic suture technique and the operation tends to take a long time. Our experience indicates that progressive practice can effectively shorten the learning time. For example, surgeons can first practice on the simulator, then practice on animal models and simple suture under laparoscopy and turn to laparoscopic gastrointestinal anastomosis in the end. In the meantime, intracorporeal hand-sewn suture can be simplified with certain novel laparoscopic tools. Knotless barbed sutures (V-Loc™; Covidien Mansfield, MA, USA) can reduce the time of anastomosis and guarantee the security of anastomosis, without involving permanent traction during the entire anastomosis procedure.

## Conclusion

In conclusion, TLTG with intracorporeal anastomosis is a secure and feasible method for the treatment of gastric cancer. With improved cosmesis, less blood loss and rapid recovery, TLTG generates favorable effects. Surgeons choose certain intracorporeal methods according to their preference and experience. Hand-sewn end-to-side esophagojejunostomy is an optimal intracorporeal anastomosis approach.

## Abbreviations

ASA: American Society of Anesthesiologists.
BMI: body mass index
EE: extracorporeal esophagojejunostomy
IE: intracorporeal esophagojejunostomy
LATG: laparoscopy-assisted total gastrectomy
LTG: laparoscopic total gastrectomy
TLTG: totally laparoscopic total gastrectomy
TNM: tumor-node-metastasis
TPN: total parenteral nutrition
